# Development and validation of a nomogram model for the prediction of 4L lymph node metastasis in thoracic esophageal squamous cell carcinoma

**DOI:** 10.3389/fonc.2022.887047

**Published:** 2022-10-03

**Authors:** Lei Xu, Jia Guo, Shu Qi, Hou-nai Xie, Xiu-feng Wei, Yong-kui Yu, Ping Cao, Rui-xiang Zhang, Xian-kai Chen, Yin Li

**Affiliations:** ^1^ Department of Thoracic Surgery, National Cancer Center/National Clinical Research Center for Cancer/Cancer Hospital, Chinese Academy of Medical Sciences and Peking Union Medical College, Beijing, China; ^2^ Department of Radiology, Beijing Shijitan Hospital, Capital Medical University, Beijing, China; ^3^ Department of Thoracic Surgery, The Affiliated Cancer Hospital of Zhengzhou University, Henan Cancer Hospital, Zhengzhou, China; ^4^ Department of Radiology, The Affiliated Cancer Hospital of Zhengzhou University, Henan Cancer Hospital, Zhengzhou, China

**Keywords:** left tracheobronchial (4L) lymph nodes, lymphadenectomy, nomogram, lymph node metastases, esophageal squamous cell carcinoma

## Abstract

**Objectives:**

The left tracheobronchial (4L) lymph nodes (LNs) are considered as regional LNs for esophageal squamous cell carcinoma (ESCC), but there is a controversy about routine prophylactic 4L LN dissection for all resectable ESCCs. This study aimed to develop a nomogram for preoperative prediction of station 4L lymph node metastases (LNMs).

**Methods:**

A total of 522 EC patients in the training cohort and 370 in the external validation cohort were included. The prognostic impact of station 4L LNM was evaluated, and multivariable logistic regression analyses were performed to identify independent risk factors of station 4L LNM. A nomogram model was developed based on multivariable logistic regression analysis. Model performance was evaluated in both cohorts in terms of calibration, discrimination, and clinical usefulness.

**Results:**

The incidence of station 4L LNM was 7.9% (41/522) in the training cohort. Patients with station 4L LNM exhibited a poorer 5-year overall survival rate than those without (43.2% vs. 71.6%, p < 0.001). In multivariate logistic regression analyses, six variables were confirmed as independent 4L LNM risk factors: sex (p = 0.039), depth of invasion (p = 0.002), tumor differentiation (p = 0.016), short axis of the largest 4L LNs (p = 0.001), 4L conglomeration (p = 0.006), and 4L necrosis (p = 0.002). A nomogram model, containing six independent risk factors, demonstrated a good performance, with the area under the curve (AUC) of 0.921 (95% CI: 0.878–0.964) in the training cohort and 0.892 (95% CI: 0.830–0.954) in the validation cohort. The calibration curve showed a good agreement on the presence of station 4L LNM between the risk estimation according to the model and histopathologic results on surgical specimens. The Hosmer–Lemeshow test demonstrated a non-significant statistic (p = 0.691 and 0.897) in the training and validation cohorts, which indicated no departure from the perfect fit. Decision curve analysis indicated that the model had better diagnostic power for 4L LNM than the traditional LN size criteria.

**Conclusions:**

This model integrated the available clinical and radiological risk factors, facilitating in the precise prediction of 4L LNM in patients with ESCC and aiding in personalized therapeutic decision-making regarding the need for routine prophylactic 4L lymphadenectomy.

## Introduction

Esophageal cancer (EC) ranks seventh in terms of cancer incidence and sixth in cancer-related mortality overall (1). Esophageal squamous cell carcinoma (ESCC) is one of the two major histological types of EC. Despite advances in screening and treatment, ESCC remains a highly lethal disease. The presence of lymph node metastases (LNMs) is one of the most important factors associated with poor prognosis (2, 3). Currently, radical esophagectomy combined with lymphadenectomy is the main treatment strategy for resectable ESCC (4). The left tracheobronchial lymph nodes (4L) are considered regional lymph nodes (LNs) for EC, but the 4L lymph node was not routinely dissected for all patients with resectable EC due to the high operative risk (5–7). The 4L lymph nodes are located deeply within the subaortic region close to the left recurrent laryngeal nerve, aortic arch, left pulmonary artery, and left main bronchial membrane, making the possibility of dissection-related damages increased and the procedure more technically challenging and time-consuming (7, 8). Therefore, a robust preoperative assessment of 4L lymph nodes is of great necessity.

Preoperative prediction of lymph node status is critical to determine the scope and method of individualized lymph node dissection for patients (9–11). Unfortunately, the current radiological assessment fails to provide reliable nodal metastasis information tailored to the individual patient. LNM is mainly assessed by imaging characteristics, including computed tomography (CT) and positron emission tomography (PET)/CT. PET/CT can significantly improve the detection of distant metastasis but shows well-known drawbacks of moderate specificity and sensitivity for the detection of locoregional node metastasis (9, 10). Moreover, many centers did not routinely perform PET/CT as the initial workup, especially in areas with poor economy or equipment. Diagnostic imaging techniques using size on CT images (>1.0 cm in short-axis diameter) as the criterion of LNM cannot exactly assess the intrathoracic nodal status in EC (11, 12). Therefore, the current radiological practice may not be adequate for the appropriate 4L LNM risk stratification of patients, and robust predictive models for preoperative assessment of nodal metastasis are of great necessity. This study aimed to develop and validate a nomogram that incorporates clinical and radiological risk factors to predict 4L LNM in ESCC and facilitate the optimal management of 4L LNs.

## Patients and methods

### Study population

A total of 892 patients who underwent radical esophagectomy and 4L lymphadenectomy were included, according to the following inclusion criteria: (1) pathological diagnosis of thoracic ESCC, (2) patients who received radical esophagectomy and 4L lymphadenectomy, (3) patients who did not receive neoadjuvant therapy, and 4) patients who underwent thoracic CT examination within 30 days before treatment. The exclusion criteria were as follows: (1) patients with adenocarcinoma, cervical EC, or gastroesophageal junction cancer; (2) patients with esophageal multiple primary cancer or other concurrent tumors; (3) patients who received neoadjuvant therapy; (4) CT data not obtained or poor image quality because of incomplete contrast or artifacts; and (5) missing information, such as age, sex, staging, and location of the tumor. Two databases of patients were used in our study. The nomogram was developed based on 522 EC patients who underwent surgery at the Department of Thoracic Surgery of Henan Cancer Hospital between January 2009 and December 2018. The external validation cohort included consisted of 370 eligible patients identified from the Department of Thoracic Surgery of National Cancer Center/Cancer Hospital from June 2018 to September 2021. This retrospective study involving human participants was reviewed and approved by the Institutional Review Board of the Chinese National Cancer Center, and informed consent was waived due to the retrospective nature of the study.

### Surgical procedures

All patients included in this study underwent transthoracic esophagectomy with 2- or 3-field lymphadenectomy. The detailed surgical procedures are described in previous reports (13, 14). During superior mediastinal LN dissection, the upper esophagus was stretched in the right anterior direction by a tape. The left main bronchus and the trachea were rotated using blade forceps in the same direction, which allowed meticulous dissection of LNs deep in the upper mediastinal region. Dissection of the 4L LNs commenced along the upper rim of the left main bronchus. The 4L LNs were dissected carefully with a narrow incision between the left main bronchus and the aortic arch. Below the aortic arch, the recurrent portion of the left recurrent laryngeal nerve was identified. The tissue, including the 4L LNs, was completely removed along the dorsal side of the left pulmonary artery. The station of the lymph node dissected was documented in detail in the surgical procedure record and each patient’s surgical records were searched. The results of 4L lymph node dissection were reconfirmed by the pathological diagnosis report of the lymph nodes. The pathological diagnosis of each lymph node was performed by two pathologists in collaboration.

### Patient characteristics, CT-reported 4L LN status and survival

The clinical characteristics included age, sex, degree of differentiation, tumor length, depth of invasion, tumor location, CT-reported 4L LN status, and short and longest axes of the largest 4L LNs. CT images were analyzed by two independent radiologists, using the picture archiving communication system (PACS). The longest axis size of 4L LN was defined as the long axis diameter of the largest 4L LN on the CT scan, and the diameter perpendicular to the long axis of the largest 4L LN was defined as the short axis (15). CT-reported positive LNs were defined as regional LNs with a short-axis diameter greater than 1.0 cm (16, 17). A central low-density mass with an irregular or rim-like enhancement of residual lymphatic tissue was considered as LN necrosis, and conglomerate nodes merging two or more nodes were considered as LN conglomeration (18–20). The overall survival (OS) was measured as the date from diagnosis to the date of death, the time of last contact, or December 2020.

### Statistical analysis

The characteristics of patients were all considered as categorical variables and were summarized using percentages. The χ2. test or Fisher’s exact test was used to compare patient characteristics between the two groups. Survival curves were generated by a Kaplan–Meier method, and the log-rank test was performed to compare differences. In addition, a propensity score matching (PSM) method was conducted to balance the baseline characteristics between the two groups. In the process of PSM, patients were matched 1:1 according to the nearest propensity score. To calculate propensity scores for each patient, a multiple logistic regression model was performed based on clinicopathological characteristics potentially affecting survival.

Univariable and multivariable logistic regression analyses were applied to identify independent risk factors of 4L LNM. Subsequently, we built the 4L LNM prediction nomogram on the basis of multivariable logistic regression analysis in the training cohort. To test the prediction ability of the nomogram model, the receiver operating characteristic (ROC) curve was performed and the prediction accuracy was evaluated through the area under the curve (AUC) in both the training cohort and the validation cohort. Additionally, calibration curves were plotted to assess the calibration of the nomogram model, accompanied by the Hosmer–Lemeshow test. The discrimination performance of the nomogram model was measured by Harrell’s concordance index (C-index) and subjected to bootstrapping validation with 1,000 resamples to calculate a relatively corrected C-index. Decision curve analysis (DCA) was used in the two cohorts to evaluate the clinical usefulness of the nomogram model. All statistical analyses were performed with R statistical software (version 3.6.0, https://cran.R-project.org). A two-sided p <0.05 was considered statistically significant.

## Results

### Patient characteristics and survival

**Figure 1:**
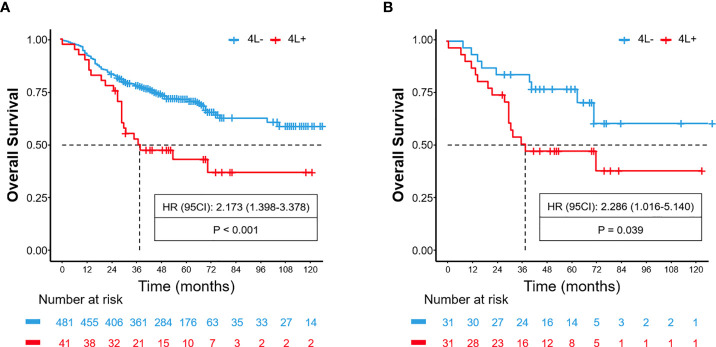
Before PSM, comparison of overall survival for patients with left tracheobronchial (4L) lymph node metastasis and non-metastasis **(A)**. After PSM, comparison of overall survival for patients with 4L lymph node metastasis and non-metastasis **(B)**.

A total of 522 patients were included in the training cohort. The clinical and radiological characteristics of all eligible patients are listed in **Tables 1**, **2**. Overall, 41 and 31 patients had 4L LNM in the training and validation cohorts, respectively. The metastasis rate was 7.9% and 8.4%, respectively (**Table 1**). As shown in **Figure 1A**, the 5-year OS rate in patients with 4L LNM was 43.2% and that in patients without 4L LNM was 71.6%. Patients with 4L LNM exhibited a poorer 5-year OS rate than those without (p < 0.001). After PSM, there was no significant difference in patient characteristics between the two groups (p > 0.05; **Table 3**). Subsequently, survival analysis showed that patients with station 4L LNM had a poorer long-term survival compared with those without (5-year OS rate: 47.2% vs. 76.9%, p = 0.039; **Figure 1B**).

**Table 1:** Baseline characteristics of patients in the training and validation cohorts.

Variables	Training cohort		Validation cohort	
	4L− (*n* = 481)	4L+ (*n* = 41)	4L− (*n* = 339)	4L+ (*n* = 31)
Age, years				
<65	257 (90.5%)	27 (9.5%)	196 (91.2%)	19 (8.8%)
≥65	224 (94.1%)	14 (5.9%)	143 (92.3%)	12 (7.7%)
Sex				
Male	311 (90.4%)	33 (9.6%)	257 (90.2%)	28 (9.8%)
Female	170 (95.5%)	8 (4.5%)	82 (96.5%)	3 (3.5%)
Smoking index				
≥400	152 (93.3%)	11 (6.7%)	90 (87.4%)	13 (12.6%)
<400	329 (91.6%)	30 (8.4%)	249 (93.3%)	18 (6.7%)
Tumor length, cm				
≤3.0	209 (91.3%)	20 (8.7%)	91 (91.9%)	8 (8.1%)
3.0 < *X* ≤ 5.0	217 (93.5%)	15 (6.5%)	130 (91.5%)	12 (8.5%)
>5.0	55 (90.2%)	6 (9.8%)	118 (91.5%)	11 (8.5%)
Tumor location				
Upper	104 (97.2%)	3 (2.8%)	38 (90.5%)	4 (9.5%)
Middle	302 (91.2%)	29 (8.8%)	133 (89.3%)	16 (10.7%)
Lower	75 (89.3%)	9 (10.7%)	168 (93.9%)	11 (6.1%)
Depth of invasion				
T1/T2	158 (97.5%)	4 (2.5%)	183 (96.8%)	6 (3.2%)
T3/T4a	323 (89.7%)	37 (10.3%)	156 (86.2%)	25 (13.8%)
Tumor differentiation				
Well/moderate	301 (95.6%)	14 (4.4%)	188 (91.3%)	18 (8.7%)
Poor	180 (87.0%)	27 (13.0%)	151 (92.1%)	13 (7.9%)
Surgical technique				
MIE	297 (90.3%)	32 (9.7%)	330 (91.7%)	30 (8.3%)
Open	184 (95.3%)	9 (4.7%)	9 (90.0%)	1 (10.0%)
Total number of resected LN				
≤15	92 (94.8%)	5 (5.2%)	13 (92.9%)	1 (7.1%)
15 < *X* ≤ 30	210 (91.7%)	19 (8.3%)	102 (91.9%)	9 (8.1%)
>30	179 (91.3%)	17 (8.7%)	224 (91.4%)	21 (8.6%)
N stage				
N0	328 (100.0%)	0 (0.0%)	166 (100.0%)	0 (0.0%)
N1	118 (92.2%)	10 (7.8%)	121 (90.3%)	13 (9.7%)
N2	27 (65.9%)	14 (34.1%)	44 (81.5%)	10 (18.5%)
N3	8 (32.0%)	17 (68.0%)	8 (50.0%)	8 (50.0%)
TNM stage				
I	72 (100.0%)	0 (0.0%)	40 (100.0%)	0 (0.0%)
II	253 (99.6%)	1 (0.4%)	146 (98.6%)	2 (1.4%)
III	147 (87.5%)	21 (12.5%)	140 (87.0%)	21 (13.0%)
IVa	9 (32.1%)	19 (67.9%)	13 (61.9%)	8 (38.1%)
Postoperative complications				
Yes	173 (91.5%)	16 (8.5%)	112 (88.2%)	15 (11.8%)
No	308 (92.5%)	25 (7.5%)	227 (93.4%)	16 (6.6%)
Postoperative adjuvant therapy				
Yes	99 (80.5%)	24 (19.5%)	178 (86.8%)	27 (13.2%)
No	382 (95.7%)	17 (4.3%)	161 (97.6%)	4 (2.4%)

LN, lymph node. 4L+ means 4L patients with lymph node metastasis. 4L- means 4L patients without lymph node metastasis.

**Table 2:** Imaging characteristics of lymph nodes in the training and validation cohorts.

Variables	Training cohort		Validation cohort	
	4L− (*n* = 481)	4L+ (*n* = 41)	4L− (*n* = 339)	4L+ (*n* = 31)
2L LNM on CT				
Yes	77 (87.5%)	11 (12.5%)	46 (80.7%)	11 (19.3%)
No	404 (93.1%)	30 (6.9%)	293 (93.6%)	20 (6.4%)
Subcarinal LNM number on CT				
0	100 (90.1%)	11 (9.9%)	271 (93.1%)	20 (6.9%)
1–2	217 (91.6%)	20 (8.4%)	68 (87.2%)	10 (12.8%)
≥3	164 (94.3%)	10 (5.7%)	0 (0.0%)	1 (100.0%)
Paraesophageal LNM number on CT				
0	244 (94.6%)	14 (5.4%)	245 (95.0%)	13 (5.0%)
1–2	136 (91.3%)	13 (8.7%)	88 (88.0%)	12 (12.0%)
≥3	101 (87.8%)	14 (12.2%)	6 (50.0%)	6 (50.0%)
Longest axis of 4L LN (cm)				
<1	381 (96.9%)	12 (3.1%)	316 (98.4%)	5 (1.6%)
1 ≤ *X* < 1.5	53 (89.8%)	6 (10.2%)	21 (67.7%)	10 (32.3%)
≥1.5	47 (67.1%)	23 (32.9%)	2 (11.1%)	16 (88.9%)
Short axis of the largest 4L LN (cm)				
<1	402 (97.1%)	12 (2.9%)	332 (97.6%)	8 (2.4%)
1 ≤ *X* < 1.2	50 (84.7%)	9 (15.3%)	5 (33.3%)	10 (66.7%)
≥1.2	29 (59.2%)	20 (40.8%)	2 (13.3%)	13 (86.7%)
4L conglomeration				
Yes	13 (48.1%)	14 (51.9%)	5 (33.3%)	10 (66.7%)
No	468 (94.5%)	27 (5.5%)	334 (94.1%)	21 (5.9%)
4L necrosis				
Yes	31 (68.9%)	14 (31.1%)	5 (25.0%)	15 (75.0%)
No	450 (94.3%)	27 (5.7%)	334 (95.4%)	16 (4.6%)
4L calcification				
Yes	38 (97.4%)	1 (2.6%)	17 (63.0%)	10 (37.0%)
No	443 (91.7%)	40 (8.3%)	322 (93.9%)	21 (6.1%)

LN, lymph node; LNM, lymph node metastasis. 4L+ means 4L patients with lymph node metastasis. 4L- means 4L patients without lymph node metastasis.

**Table 3:** Clinical characteristics of patients in the training cohorts after PSM.

Variables	4L− (*n* = 31)	4L+ (*n* = 31)	*p*-value
Age, years			0.796
<65	19 (51.4%)	18 (48.6%)	
≥65	12 (48.0%)	13 (52.0%)	
Sex			0.224
Male	22 (45.8%)	26 (54.2%)	
Female	9 (64.3%)	5 (35.7%)	
Smoking index			0.576
≥400	8 (44.4%)	10 (55.6%)	
<400	23 (52.3%)	21 (47.7%)	
Tumor location			0.861
Upper	4 (57.1%)	3 (42.9%)	
Middle	21 (51.2%)	20 (48.8%)	
Lower	6 (42.9%)	8 (57.1%)	
Depth of invasion			0.335
T1/T2	8 (25.8%)	4 (12.9%)	
T3/T4a	23 (74.2%)	27 (87.1%)	
Tumor differentiation			0.127
Well/moderate	19 (59.4%)	13 (40.6%)	
Poor	12 (40.0%)	18 (60.0%)	
Surgical technique			0.776
MIE	23 (51.1%)	22 (48.9%)	
Open	8 (47.1%)	9 (52.9%)	
N stage			0.421
N1	15 (60.0%)	10 (40.0%)	
N2	9 (45.0%)	11 (55.0%)	
N3	7 (41.2%)	10 (58.8%)	
TNM stage			0.423
II	1 (50.0%)	1 (50.0%)	
III	23 (56.1%)	18 (43.9%)	
IVa	7 (36.8%)	12 (63.2%)	
Postoperative complications			0.421
Yes	9 (42.9%)	12 (57.1%)	
No	22 (53.7%)	19 (46.3%)	
Postoperative adjuvant therapy			0.303
Yes	16 (44.4%)	20 (55.6%)	
No	15 (57.7%)	11 (42.3%)	

4L+ means 4L patients with lymph node metastasis. 4L- means 4L patients without lymph node metastasis.

### Development of the nomogram model

**Figure 2:**
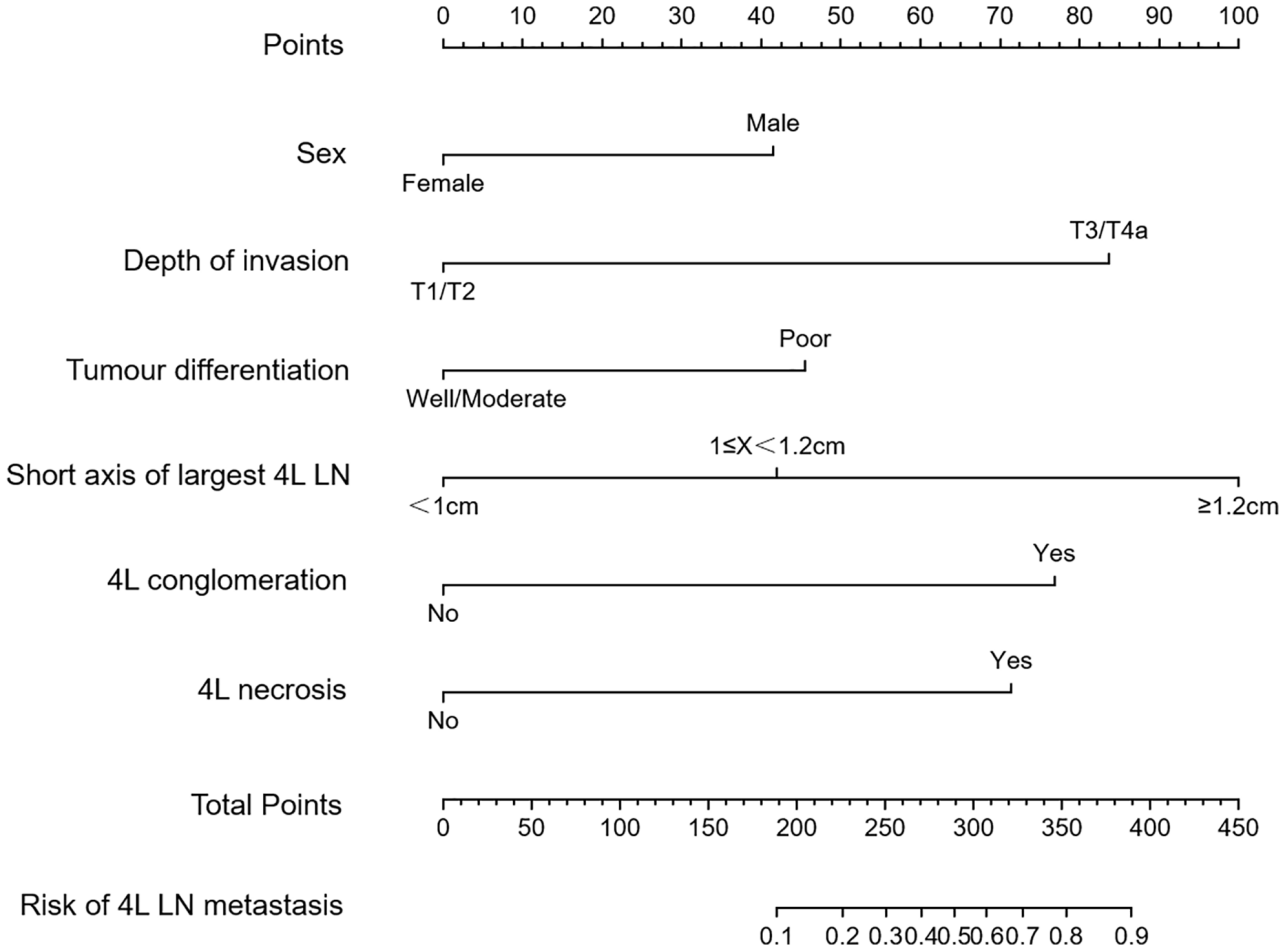
Nomogram model for predicting 4L lymph node metastasis (LNM) in patients with thoracic ESCC. The instructions were as follows: locate one patient’s characteristics on the corresponding axis to determine how many points the patient receives, add up the total number of points and locate this point on the total points axis, and draw a vertical line to identify the patient’s probability of 4L LNM.

Univariate and multivariate logistic regression analyses were performed to identify the independent risk factors associated with 4L LNM (**Table 4**). Seven variables were confirmed as independent 4L LNM risk factors: sex (odds ratio [OR] = 2.864, 95% confidence interval [CI]: 1.052–7.795, *p* = 0.039), depth of invasion (OR = 5.464, 95% CI: 1.566–19.062, *p* = 0.002), tumor differentiation (OR = 2.790, 95% CI: 1.226–6.347, *p* = 0.016), short axis of the largest 4L LN (OR = 3.096, 95% CI: 1.106–8.668, *p* = 0.031; OR = 18.675, 95% CI: 7.664–45.507, *p* < 0.001), 4L conglomeration (OR = 5.534, 95% CI: 1.621–18.885, *p* = 0.006), and 4L necrosis (OR = 3.701, 95% CI: 1.452–9.432, *p* = 0.002). The above independent predictors were incorporated to develop a nomogram for predicting the probability of 4L LNM (**Figure 2**).

**Table 4:** Univariate and multivariate analyses for 4L lymph node metastasis in the training cohort.

Variables	Univariate analysis		Multivariate analysis	
	OR (95% CI)	*p*-value	OR (95% CI)	*p*-value
Age, years	1.681 (0.860–3.285)	0.129		
Sex	2.255 (1.019–4.992)	0.045	2.864 (1.052–7.795)	0.039
Smoking index	0.794 (0.387–1.626)	0.528		
Tumor length, cm		0.554		
≤3.0	Reference			
3.0 < *X* ≤ 5.0	1.140 (0.437–2.976)	0.789		
>5.0	1.578 (0.585–4.255)	0.367		
Tumor location		0.053		0.642
Upper	Reference		Reference	
Middle	0.240 (0.063–0.918)	0.037	1.847 (0.413–8.273)	0.771
Lower	0.300 (0.090–1.007)	0.051	1.747 (0.472–6.466)	0.656
Depth of invasion	4.525 (1.585–12.917)	0.005	5.464 (1.566–19.062)	0.002
Tumor differentiation	0.310 (0.185–0.607)	0.001	2.790 (1.226–6.347)	0.016
Total number of resected LN		0.550		
≤15	Reference			
15 < *X* ≤ 30	1.747 (0.625–4.887)	0.287		
> 30	1.050 (0.530–2.080)	0.889		
2L LNM on CT	1.924 (0.925–4.002)	0.080	1.869 (0.760–4.599)	0.164
Subcarinal LNM number on CT		0.407		
0	Reference			
1–2	0.554 (0.227–1.352)	0.195		
≥3	0.662 (0.302–1.451)	0.303		
Paraesophageal LNM number on CT		0.081		0.220
0	Reference		Reference	
1–2	2.416 (1.112–5.250)	0.026	1.701 (0.667–4.338)	0.687
≥3	1.450 (0.653–3.220)	0.361	2.111 (0.810–5.497)	0.151
Longest axis of 4L LN (cm)		<0.001		0.379
<1	Reference		Reference	
1 ≤ *X* < 1.5	15.537 (7.260–33.253)	<0.001	1.690 (0.520–5.892)	0.194
≥ 1.5	4.323 (1.621–11.524)	0.003	0.921 (0.370–1.644)	0.337
Short axis of the largest 4L LN (cm)		<0.001		0.001
<1	Reference		Reference	
1 ≤ *X* < 1.2	23.103 (10.290–51.874)	<0.001	3.096 (1.106–8.668)	0.031
≥1.2	3.831 (1.542–9.519)	0.004	18.675 (7.664–45.507)	<0.001
4L conglomeration	18.667 (7.988–43.619)	<0.001	5.534 (1.621–18.885)	0.006
4L necrosis	7.527 (3.587–15.792)	<0.001	3.701 (1.452–9.432)	0.002
4L calcification	0.291 (0.039–2.179)	0.230		

OR, odds ratio; 95% CI, 95% confidence interval; LN, lymph node; LNM, lymph node metastasis. 4L+ means 4L patients with lymph node metastasis. 4L- means 4L patients without lymph node metastasis.

### Internal and external validations

**Figure 3:**
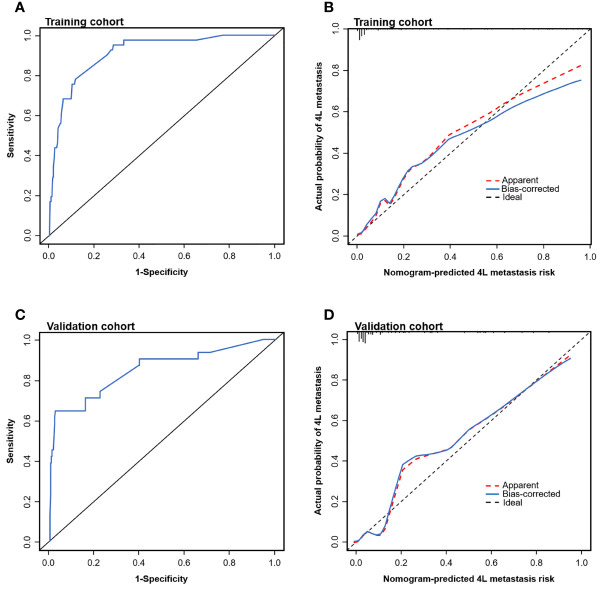
Evaluation of the nomogram model for predicting 4L LNM. Receiver operating characteristic (ROC) curves to discriminate 4L LNM (+) from 4L LNM (−) for the nomogram model in the training cohort **(A)** and the validation cohort **(C)**. Calibration curves of the nomogram model in the training cohort **(B)** and the validation cohort **(D)**. Calibration curves depicted the calibration of the nomogram model in terms of agreement between the predicted risk and observed outcomes of 4L LNM.

To test the prediction ability of the nomogram model, internal and external validations were conducted. The AUC of the nomogram that was constructed on the basis of the training data set was 0.911 (95% CI: 0.869–0.954), demonstrating a good discrimination of 4L LNM (**Figure 3A**). The calibration curve showed good agreement on the presence of 4L LNM between the risk estimation according to the model and histopathologic results on surgical specimens (**Figure 3B**). The Hosmer–Lemeshow test demonstrated a non-significant statistic (p = 0.691), which indicated no departure from the perfect fit.

**Figure 4:**
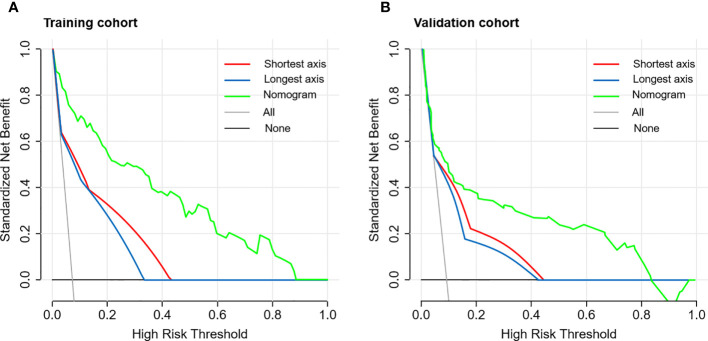
The decision curve analysis (DCA) of the nomogram model for predicting 4L LNM in the training cohort **(A)** and the validation cohort **(B)**. The red line represented the prediction based on the short axis of the largest 4L LNs alone. The blue line represented the prediction based on the longest axis of the largest 4L LNs alone. The green line represented the nomogram model. Across the majority of the range of risk thresholds, the nomogram model had the highest net benefit compared with the prediction based on either the longest axis or the short axis of the largest 4L LNs alone.

External validation was conducted in an independent cohort. It was then applied to the nomogram and produced an AUC of 0.851 (95% CI: 0.765–0.937), suggesting a good performance of our prediction model (**Figure 3C**). The calibration curve showed good agreement between the actual and predicted risks of 4L LNM (**Figure 3D**). The Hosmer–Lemeshow test yielded a p-value of 0.897, indicating a good fit. The DCA for the nomogram model and the models based on CT-reported short or longest axis of the largest 4L LNs is presented in **Figure 4**. The DCA indicated that, across the majority of the range of risk thresholds, using the nomogram model to predict 4L LNM added the net benefit, and within this range, the highest net benefit emerged with the nomogram model compared with the models of either the longest axis of the largest 4L LNs on CT or the short axis alone. Similar results were obtained in both the training and validation cohorts.

## Discussion

Regional lymph node metastasis is a negative prognostic factor in patients with EC (2, 3). 4L LNs are defined as regional LNs according to the UICC/AJCC TNM classification (8th edition) and the Japanese Classification of Esophageal Cancer (10th edition) (6, 7). This study found that the 4L LNM rate was 7.4% in the training group, which was comparable to some previous studies (7, 21). The survival benefit received from 4L lymph node dissection in patients with ESCC has been poorly studied, mainly because of the low metastasis rate of the 4L LNs and the limited number of patients receiving the 4L LN dissection (5, 7). This study showed that in the training group, 4L LNM was associated with a poorer prognosis. Currently, there was still controversy about routine prophylactic 4L LN dissection, due to complex adjacent structures and low metastasis rate (7–9). We developed and validated a nomogram model for the individualized prediction of 4L LNM in patients with ESCC. The nomogram model could successfully stratify patients according to their risk of 4L LNM and a 4L lymphadenectomy was performed according to the 4L LNM risk stratification.

Currently, the discrimination of malignant from benign LNs in esophageal cancer with traditional practice based on clinical characteristics remains challenging. In this study, the nomogram model incorporated six clinical and radiological factors based on multivariable logistic regression analysis. Univariate analysis of a previous study found a difference in LNM rates between men and women with esophageal cancer (22). Our study found that male patients showed a higher rate of 4L LNM than female patients. The depth of tumor invasion, as a risk factor for LNM in patients with early ESCC, was supported by some studies (23, 24). Similarly, our results also showed that the depth of invasion was an independent risk factor for 4L LNM. In addition, the grade of differentiation was strongly predictive of LNM according to several reports (24, 25). Tumor differentiation was identified as an independent predictive factor for 4L LNM in this study. Therefore, the grade of differentiation was included during the development of the nomogram model.

In clinical practice, preoperative diagnosis of LNM was mainly based on various imaging methods. The detection of LNM on CT images mainly depended on size. In general, intrathoracic and abdominal LNs with a diameter greater than 1.0 cm were considered to be enlarged (15–17). Multivariable analysis in this study showed that the short axis of the largest 4L LNs on imaging was an independent predictive factor for 4L LNM. Similarly, a study reported that the short axis was independently associated with recurrent laryngeal nerve LNM (26). However, unlike the short axis, the longest axis of the largest 4L LNs was not identified as a risk factor in the multivariable analysis, which may be due to confounding caused by other predictive factors, such as the short axis of the largest LNs. Of note, a study suggested that lymph node necrosis was a risk predictor of cervical LNM in patients with head and neck squamous cell carcinoma (18). This was consistent with our results showing that lymph node necrosis and conglomeration were associated with the increased rate of 4L LNM in ESCC. The reason may be that they are the behavioral characteristics of LNM on imaging.

Yokota and colleagues (27) indicated that clinical node diagnosis had lower specificity and little predictive value in the preoperative diagnosis of LNM for patients with locally advanced EC. Some studies used the common size criterion of 1.0 cm in CT-reported enlarged nodes, but it showed a low prediction accuracy (13, 14). In addition, the presence of inflammatory and benign enlarged LNs reduced the specificity further. A nomogram was used to help answer a focused clinical question and aid in clinical decision-making. Our study developed the first nomogram model for the prediction of 4L LNM incorporating seven predictive clinical and imaging factors. The DCA indicated that the nomogram model had better diagnostic power for 4L LNM than the traditional LN size criteria, including the models of either the longest axis of the largest 4L LNs on CT images or the short axis alone. Therefore, combined with the results of validation analyses, we believed that our nomogram model could aid in accurate diagnosis and therapeutic decision-making in 4L lymphadenectomy tailored to individual patients with ESCC. PET/CT, ultrasound-guided bronchoscopy with fine-needle aspiration (EBUS-FNA), and endoscopic esophageal ultrasound-guided fine-needle aspiration (EUS-FNA) had certain advantages in distinguishing malignant and benign lymph nodes, compared with chest CT. Some studies suggested the potential diagnostic roles of PET/CT and EBUS-FNA in detecting LNM (28–30). However, EBUS-FNA and EUS-FNA had high specificity but low negative predictive value and were invasive techniques (31). Moreover, many centers did not routinely perform PET/CT and EBUS-FNA as the initial workup, especially in areas with poor economy or equipment. Due to the limited number of patients receiving PET/CT and EBUS-FNA examinations, we did not include PET/CT and EBUS-FNA assessment in the analysis. In clinical practice and decision-making, for patients who have not received PET/CT and/or EBUS-FNA examination, this model can aid in the prediction of 4L LNM. For patients receiving PET/CT and/or EBUS-FNA, this model can further confirm the results of PET/CT and/or EBUS-FNA examination. Further studies may be needed to reveal whether adding LN characteristics in PET/CT and EBUS-FNA examinations to this nomogram model could improve the predictive efficacy for 4L LNM.

Our study has limitations. Firstly, this is a retrospective study. Excluded patients who did not undergo 4L lymphadenectomy, the interobserver bias among radiologists, and inhomogeneity of surgical techniques still could bias the results. Secondly, this study focused only on the binary classification of patients according to the presence of 4L LNM regardless of the actual quantity. The efficacy of the nomogram model in the prediction of the detailed number of 4L LNM should be verified in further studies. Finally, only CT-reported 4L LN status and short and longest axes of the largest 4L LNs were included in this study. More imaging features, such as CT value, will expand the feature pool, which may result in more valuable factors being identified. Further studies are needed to confirm our findings.

## Conclusions

This study developed a nomogram model for the prediction of 4L LNM that was negatively associated with the prognosis of patients with thoracic ESCC. This model that integrated preoperatively the available clinical and radiological risk factors could become an effective tool in predicting 4L LNM and aid in personalized therapeutic decision-making regarding the need for 4L lymphadenectomy.

## Data availability statement

The original contributions presented in the study are included in the article/supplementary material. Further inquiries can be directed to the corresponding authors.

## Ethics statement

The Institutional Review Board of Chinese National Cancer Center has reviewed our clinical project, and supported us to conduct the study. This retrospective study was reviewed, and on file, and approved by the Institutional Review Board of Chinese National Cancer Center, and the informed consents were waived, due to the retrospective nature.

## Author contributions

Conception and design: All authors. Provision of study materials or patients: LX, JG, H-nX, X-fW, Y-kY, X-kC, and YL. Collection and assembly of data: LX, JG, H-nX, X-fW, Y-kY, PC, R-xZ, X-kC, and YL. Data analysis and interpretation: All authors. Manuscript writing: All authors. Final approval of manuscript: All authors. Accountable for all aspects of the work: All authors.

## Funding

This study was funded by the Special Program for Basic Resource Survey of the Ministry of Science and Technology (2019FY101101).

## Conflict of interest

The authors declare that the research was conducted in the absence of any commercial or financial relationships that could be construed as a potential conflict of interest.

## Publisher’s note

All claims expressed in this article are solely those of the authors and do not necessarily represent those of their affiliated organizations, or those of the publisher, the editors and the reviewers. Any product that may be evaluated in this article, or claim that may be made by its manufacturer, is not guaranteed or endorsed by the publisher.
